# Case Report: Perianal infection in children caused by ingested jujube pits: a report of two cases

**DOI:** 10.3389/fped.2024.1379689

**Published:** 2024-04-10

**Authors:** Guoping Jiang, Lixu Wu, Weiwei Ruan, Qian Shao, JianMing Zhu

**Affiliations:** ^1^ Department of Pediatric Surgery, Ningbo Women and Children's Hospital, Ningbo, China; ^2^ School of Medicine, Ningbo University, Ningbo, China

**Keywords:** perianal, infection, children, ingested, jujube pit, case report

## Abstract

**Background:**

The ingestion of jujube pits by children is a rare cause of perianal infection.This article aimed to report two cases of perianal infection in children resulting from the ingestion of jujube pits.

**Methods:**

We reviewed the clinical records of perianal infection caused by jujube pits at our hospital. Details of the patients’ presentation, imaging studies, complications and treatment were recorded.

**Results:**

Both pediatric patients presented with perianal swelling and pain. The caregivers of both patients denied a history of jujube consumption. Magnetic resonance imaging (MRI) indicated the presence of jujube pits, which were subsequently removed during surgery. Postoperatively, both patients recovered well, and follow-up showed no recurrence or the formation of anal fistulas.

**Conclusion:**

The ingestion of jujube pits leading to perianal infection is rare and inconspicuous. Early diagnosis and treatment are beneficial in preventing the occurrence of serious complications.

## Introduction

This article reports two cases of perianal infection in children resulting from the ingestion of jujube pits. In both cases, the pits became lodged in the anal canal, gradually shifting outward, leading to infection in the perianal tissues. Both cases presented with symptoms of perianal infection, with swelling evident on digital rectal examination but without apparent foreign bodies or fistulas. MRI confirmed the presence of perianal infection and identified the jujube pits. Extraction of the pits under general anesthesia was performed, resulting in successful recovery. No recurrence or anal fistula was observed during follow-up. The diagnosis and management of perianal infections in children should not overlook the rare occurrence of jujube pit impaction. Early diagnosis and appropriate treatment are crucial for alleviating pain and minimizing the risk of serious complications.

## Case reports

**Figure 1:**
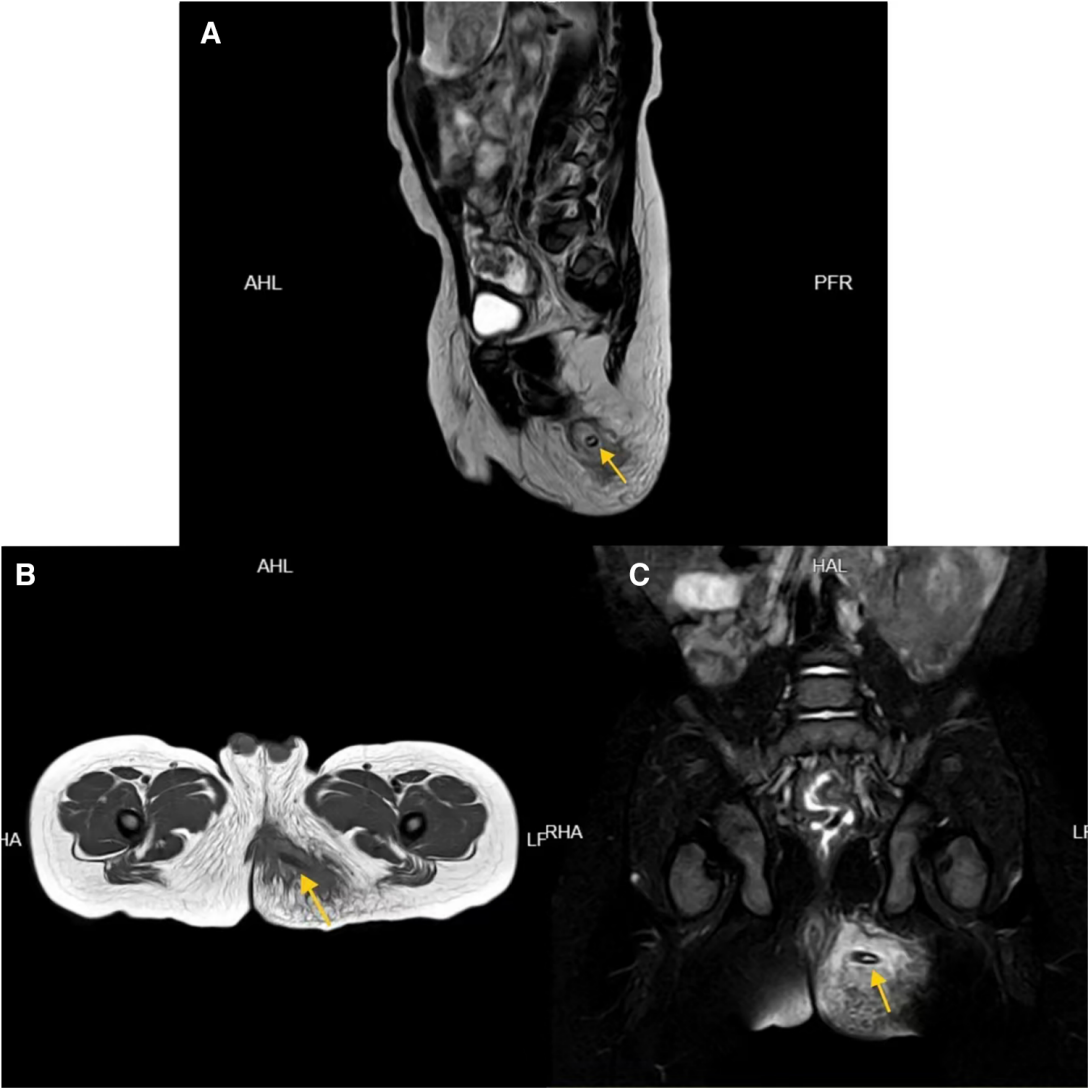
MRI images of patient 1 in the sagittal (**A**), horizontal (**B**), and coronal plane (**C**) showing the jujube pit (indicated by arrows).

**Figure 2:**
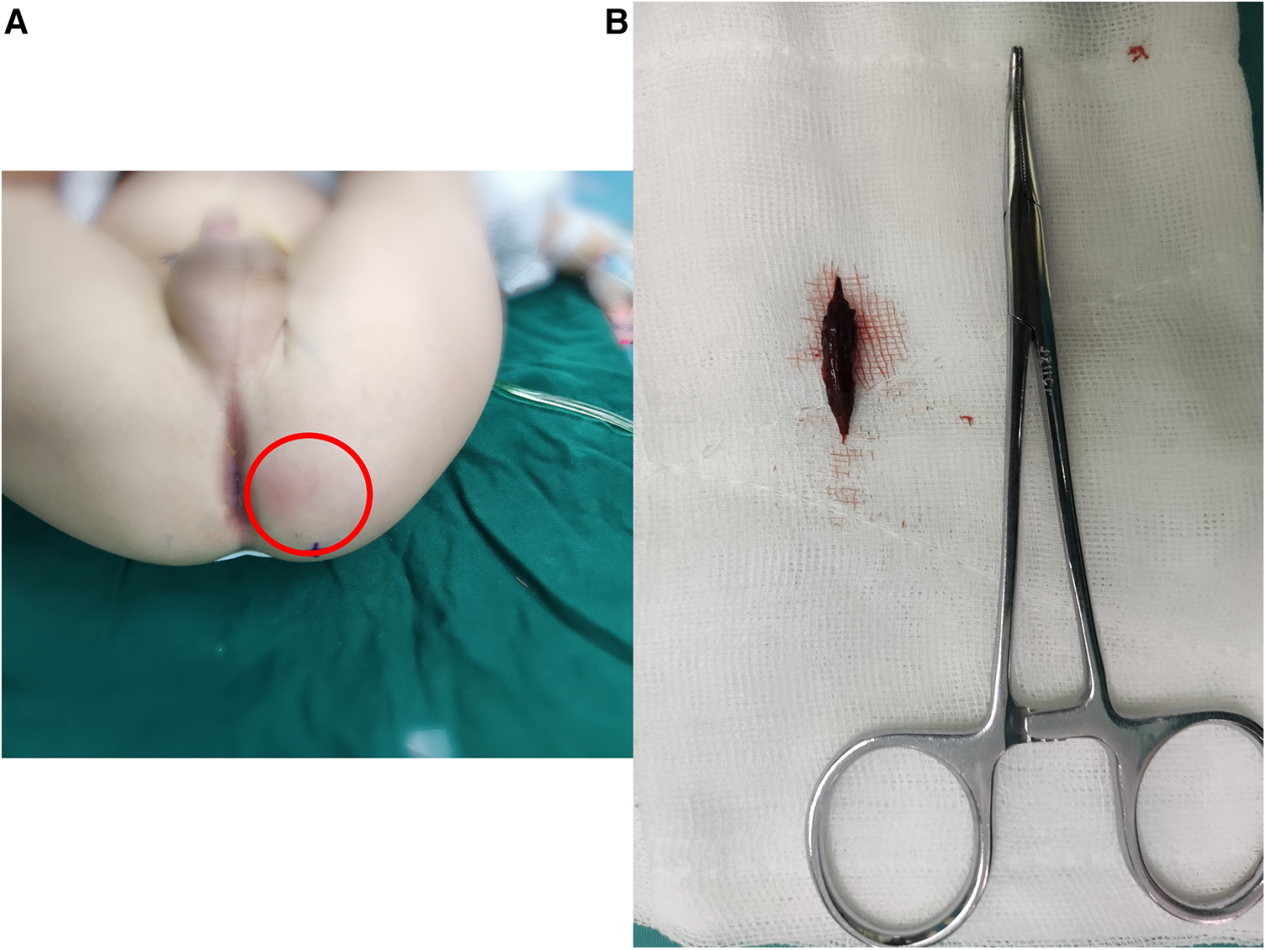
Perianal infection area in patient 1 with a relatively large extent indicated by the red circle (**A**), and the extracted jujube pit after surgery (**B**).

Case 1: A 16-month-old male with left buttock redness and pain persisting for two weeks. The patient also experienced diarrhea and fever. Initial treatment with topical ointments and oral antibiotics yielded no improvement. Subsequent examination revealed a swollen and tender area on the left buttock. Rectal examination revealed swelling in the rectum but no evidence of foreign bodies or fistulas. MRI confirmed jujube pit impaction in the left buttock (Figure 1). After antibiotic treatment and bowel preparation, the jujube pit was successfully removed under general anesthesia (Figure 2). The wound was irrigated, a negative-pressure drain was placed, and the patient recovered and was discharged 10 days later, with no recurrence observed at the 1-year-4-month follow-up.

**Figure 3:**
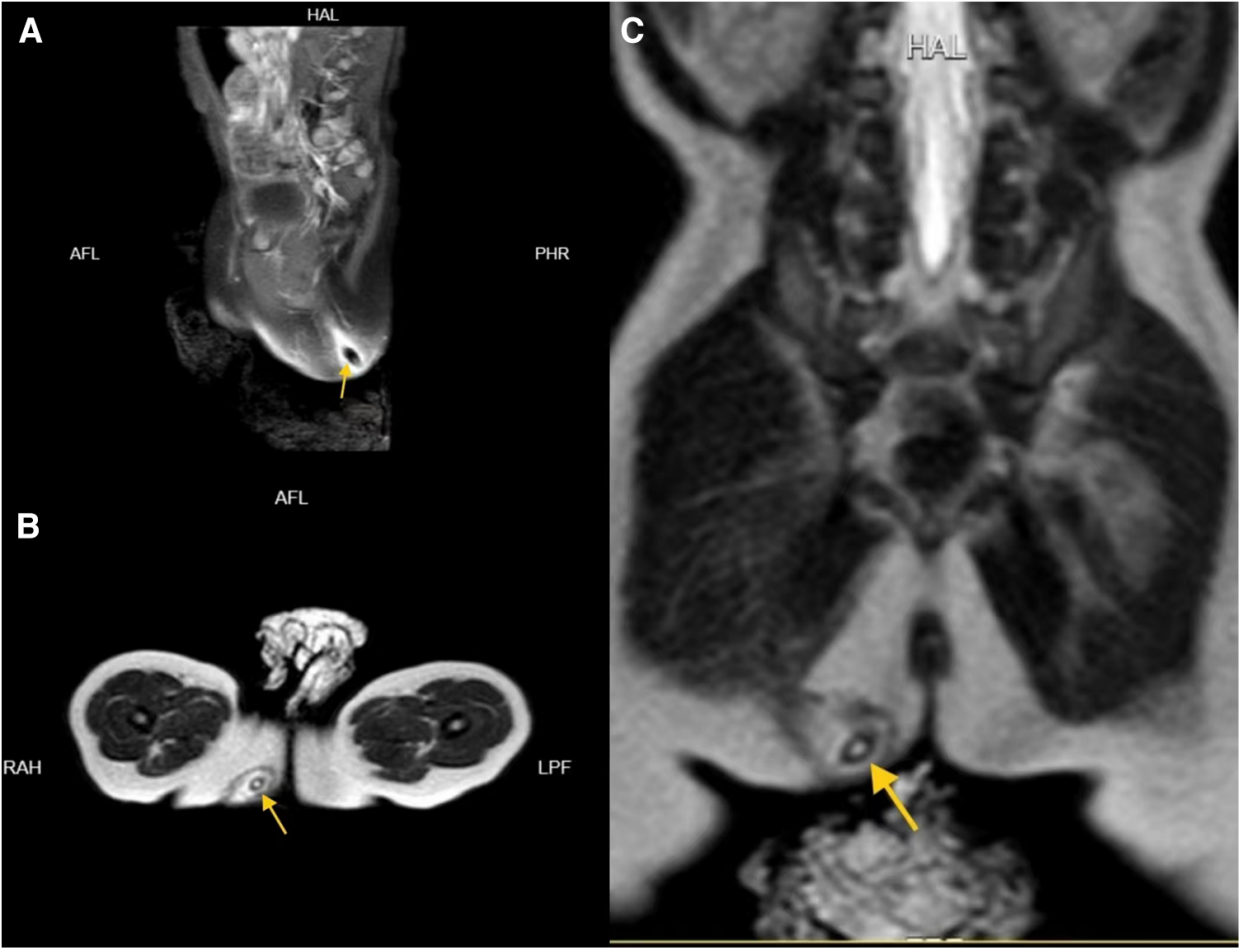
MRI images of patient 2 in the sagittal (**A**), horizontal (**B**), and coronal plane (**C**) showing the jujube pit (indicated by arrows).

Case 2: A 1-year-old male without significant medical history presented with perianal pain and discomfort. He developed right buttock redness and pain associated with fever. Initial treatment with oral antibiotics and topical antimicrobial agents showed no improvement. Examination revealed a swollen and tender area on the right buttock.Rectal examination revealed swelling but no foreign bodies or fistulas. MRI confirmed jujube pit impaction in the right buttock (Figure 3). After antibiotic treatment and bowel preparation, the jujube pit was successfully removed under general anesthesia. The wound was irrigated, a negative-pressure drain was placed, and the patient recovered well and was discharged 11 days later, with no recurrence observed at the 2-month follow-up.

## Discussion

Ingesting foreign bodies into the digestive tract is a common emergency situation in children, with approximately 80%–90% naturally passing out of the body (1). However, special objects (such as magnets) or sharp objects (including jujube pits) indeed carry a high risk of mucosal injury in the digestive tract. Adverse events may include perforation, abscess, fistula, peritonitis, sepsis, or even death (2). It is relatively rare for jujube pits to become lodged in the anal canal, leading to perianal infection. Literature reports indicate that jujube pits most commonly get lodged in the esophagus, followed by the pylorus and duodenum (3), with the anal canal being an extremely rare site for jujube pit impaction (4). Due to the narrowness of the anal canal and the contraction of the anal sphincter, sharp and hard foreign bodies are more likely to become lodged, causing penetration into surrounding tissues and triggering infection.

Children's oral tendencies, especially during the oral stage, increase the risk of ingesting brightly colored jujubes. Communication challenges may obscure the medical history, posing a risk of overlooking rare causes like jujube pit impaction in perianal infections. In the two cases presented in this article, caregivers initially denied any history of jujube ingestion, but other family members confirmed jujube consumption. Additionally, both patients exhibited symptoms such as diarrhea and perianal discomfort, which may be related to jujube pit impaction in the anal canal. Therefore, primary prevention is crucial, aiming to prevent children from coming into contact with potentially harmful objects.

For cases where jujube pit impaction has already led to perianal infection, the unclear medical history and the unique texture of jujube pits make routine digital rectal examination and x-ray ineffective. CT and MRI imaging not only detect the presence and location of jujube pit foreign bodies but also assess damage to adjacent tissues (5). The treatment approach is well-established. We recommend ultrasound-guided incision and removal of the foreign body under general anesthesia, with careful inspection for any injuries or fistula formation. Thorough irrigation of the wound and appropriate drainage placement are essential. Notably, both cases presented with a relatively extensive area of infection. Surgery revealed minimal purulent fluid, likely due to the short symptom duration. The process of jujube pit impaction migrating from the anal canal to the perianal tissues, resembling the scenario in anal fistula procedures with seton placement, may explain the absence of perianal fistula formation.

## Conclusion

In conclusion, perianal infection in children caused by ingested jujube pit impaction is extremely rare. A relatively extensive area of infection, along with a history of jujube consumption, and symptoms such as diarrhea, perianal discomfort, or recurrent infections may raise suspicions for the diagnosis. We recommend early confirmation through MRI or CT imaging and prompt surgical removal of the jujube pit, which is crucial for effective treatment.

## Data Availability

The original contributions presented in the study are included in the article/Supplementary Material, further inquiries can be directed to the corresponding author.
